# Spatiotemporal Gait Parameters and Static Balance Across Locomotor Conditions in Young Adults with Unilateral Flatfoot: A Cross-Sectional Comparative Study

**DOI:** 10.3390/jcm15207973

**Published:** 2026-10-15

**Authors:** Geun-Hyung Park, Ju-Yeon Jung, Hwi-Young Cho, Eui-Young Jung

**Affiliations:** 1 Department of Physical Therapy, Gachon University, Incheon 21936, Republic of Korea; 2 Institute of Human Convergence Health Science, Gachon University, Incheon 21936, Republic of Korea; 9955me@gachon.ac.kr

**Keywords:** flatfoot, gait analysis, postural balance, walking, running

## Abstract

**Background/Objectives:** Flatfoot (FF) may alter gait and postural control, but whether these differences vary across different locomotor conditions remains unclear. We compared gait and static balance in young adults with and without unilateral FF across locomotor conditions. **Methods:** This cross-sectional comparative study included 28 young adults classified into a unilateral FF group (*n* = 14) or a control group (*n* = 14). Spatiotemporal gait parameters, the primary outcomes, were assessed on an instrumented treadmill during preferred speed walking, running at 9 km/h, and uphill and downhill walking at ±10% grades. Static balance was assessed using center of pressure (COP) measures. Gait parameters were analyzed using a 2 × 4 repeated-measures ANOVA. **Results:** Group × condition interactions were significant for the involved single support phase (*p* = 0.042) and step width (*p* = 0.010). The involved single support phase was lower in the FF group during preferred speed walking (*p* = 0.003), and step width was greater during the three walking conditions (all *p* < 0.001) but not during running. Averaged across conditions, the FF group showed a greater uninvolved stance phase (*p* = 0.017) and shorter step and stride lengths (all *p* < 0.05). COP path length was greater in the FF group (*p* = 0.005). **Conclusions:** Gait differences in unilateral FF were observed across specific gait parameters and locomotor conditions rather than as a uniform pattern. These exploratory findings suggest that considering multiple locomotor conditions and both limbs may provide additional descriptive information when characterizing gait in unilateral FF.

## 1. Introduction

Flatfoot (FF) is characterized by a reduction in the medial longitudinal arch and increased foot pronation during weight bearing [1,2]. Static foot posture associated with FF can be assessed using the Foot Posture Index-6 (FPI-6), which evaluates multiple components of foot posture during standing [3]. The prevalence of flatfoot decreases during skeletal growth, and has been reported in approximately 11% of individuals aged 18–25 years [4]. Although FF may be asymptomatic, alterations in foot structure and pronation can modify the alignment and mechanical behavior of the foot–ankle complex, potentially affecting shock absorption, weight bearing, propulsion, and postural control during functional activities [5]. Consistent with this, flatfoot has been associated with lower-limb musculoskeletal injuries [6,7].

Altered gait characteristics have been reported in individuals with FF compared with individuals with a normal foot posture. FF has been associated with differences in stride length and gait speed, as well as altered ankle and hip kinematics [8]. Multi-segment foot analyses have further demonstrated greater hindfoot eversion and forefoot abduction [9], while differences in plantar-pressure distribution and center-of-pressure patterns have also been reported during walking [10,11]. These alterations in foot motion and plantar loading may be accompanied by differences in spatiotemporal gait characteristics, which quantify the timing and spatial organization of successive steps. However, these findings have been derived predominantly from level walking, providing limited insight into gait behavior under more demanding locomotor conditions.

Previous studies reported that walking on inclined surfaces alters lower-limb intersegmental coordination and spatiotemporal gait parameters [12,13]. In individuals with FF, ascending-slope walking has been associated with altered lower-extremity kinematics compared with those with a normal foot posture [14], whereas gait alterations during downhill walking remain less well characterized in this population. During running, individuals with FF have also demonstrated distinct rearfoot, midfoot, and forefoot kinematics [15]. However, inclined walking and running have largely been examined in separate studies. In addition, unilateral FF warrants separate consideration from bilateral FF because only one limb presents an altered foot posture, while the contralateral limb retains a normal foot posture. Previous studies have reported side-to-side neuromuscular differences in unilateral FF [16] and differences in postural alignment between unilateral and bilateral FF [17]. Evidence therefore remains limited regarding how the spatiotemporal gait parameters of the FF side and the contralateral side in individuals with unilateral FF differ from those observed in individuals with a normal foot posture across level walking, uphill walking, downhill walking, and running within the same participants. Differences in postural stability have also been reported in individuals with FF [18,19,20]. Because gait and static balance reflect different aspects of motor function, assessing both may provide complementary information in individuals with FF.

Accordingly, this study aimed to compare the spatiotemporal gait parameters of the FF side and the contralateral side across four locomotor conditions, as well as static balance, between adults with unilateral FF and those with a normal foot posture. We hypothesized that the unilateral FF group would exhibit different spatiotemporal gait parameters, with between-group differences varying across locomotor conditions. In particular, we expected spatiotemporal gait differences on the FF side to become more pronounced during uphill walking, downhill walking, and running than during level walking. We further hypothesized that the unilateral FF group would demonstrate greater postural sway during static standing, reflected in a greater COP sway area, path length, and sway velocity.

## 2. Materials and Methods

### 2.1. Study Design

A cross-sectional comparative design was adopted, with all assessments performed at Gachon University. The protocol received approval from the Gachon University Institutional Review Board (No. 1044396-202605-HR-158-01; July 2026), and the study adhered to the Declaration of Helsinki. The study was registered with the Clinical Research Information Service (CRIS) of the Republic of Korea (registration number: KCT0012442; 12 August 2026). Written informed consent was obtained from all participants before enrollment.

### 2.2. Participants

Participants were recruited in July 2026 through bulletin board advertisements at Gachon University and social networking services. A total of 30 individuals were screened for eligibility, and 28 eligible participants completed the protocol. The general inclusion criteria were as follows: (1) aged 20 years or older and (2) able to complete both treadmill walking and treadmill running at 9 km/h without lower-limb pain. The exclusion criteria were as follows: (1) a body mass index (BMI) of 30 kg/m^2^ or higher; (2) a history of lower-extremity or spinal surgery within six months; (3) an orthopedic condition of the foot, ankle, knee, hip, or spine likely to alter gait; (4) a neurological or vestibular disorder likely to affect balance or gait; (5) an acute injury or inflammatory condition of the lower limb; and (6) bilateral flatfoot, defined as an FPI-6 score ≥ 6 on both feet [10,21]. Foot posture was assessed bilaterally using the FPI-6 by a single physical therapist with 7 years of clinical experience. Each foot was assessed twice, and the mean of the two measurements was used for analysis. The reliability of the FPI-6 has been reported previously [22,23]. According to established FPI-6 reference values, scores of ≥6 indicate a pronated foot posture. In this study, this threshold was used as the operational criterion for unilateral flatfoot, defined as an FPI-6 score of ≥6 on one foot and <6 on the contralateral foot [3,22]. Accordingly, the terms flatfoot and FF in this manuscript refer to this FPI-6-based classification rather than a confirmed structural diagnosis. Participants with FPI-6 scores of <6 on both feet were classified into the control group.

Sample size estimation was performed using G*Power (version 3.1.9.7; Heinrich Heine University, Düsseldorf, Germany). Because no directly comparable study had evaluated flatfoot and control groups across four locomotor conditions, a medium effect size was assumed [24] for the group × condition interaction in spatiotemporal gait outcomes. The calculation used an F test for a repeated-measures ANOVA with a within–between interaction, specifying an effect size of f = 0.25, α = 0.05, statistical power of 0.80, two groups, four repeated measurements, a correlation of 0.5 among repeated measures, and a nonsphericity correction of ε = 1, with the correlation and nonsphericity correction set to the software default values. This analysis indicated that at least 24 participants were required, corresponding to 12 participants per group. To allow for an anticipated 20% attrition rate, 30 individuals were initially targeted for enrollment.

### 2.3. Procedures

All measurements were completed in a laboratory at Gachon University. Anthropometric measurements were obtained before the experimental assessments. All participants were tested on an instrumented treadmill (FDM-T; Zebris Medical GmbH, Isny, Germany) while wearing standardized socks.

Preferred walking speed was determined using the ascending–descending protocol described by Dal et al. [25]. Belt speed was increased in increments of 0.1 km/h from a low starting speed until the participant identified the speed at which walking felt most comfortable. The belt speed was then increased above the selected speed and subsequently decreased in increments of 0.1 km/h until the participant again identified the most comfortable walking speed. The preferred walking speed was calculated as the mean of the speeds identified during the ascending and descending trials. Participants then completed a 5-min warm-up and familiarization period, walking on the treadmill at their preferred walking speed. Participants were then familiarized with treadmill running at 9 km/h for 1 min to ensure that the running condition could be performed safely and consistently. A 5-min rest period was provided following familiarization.

**Figure 1:**
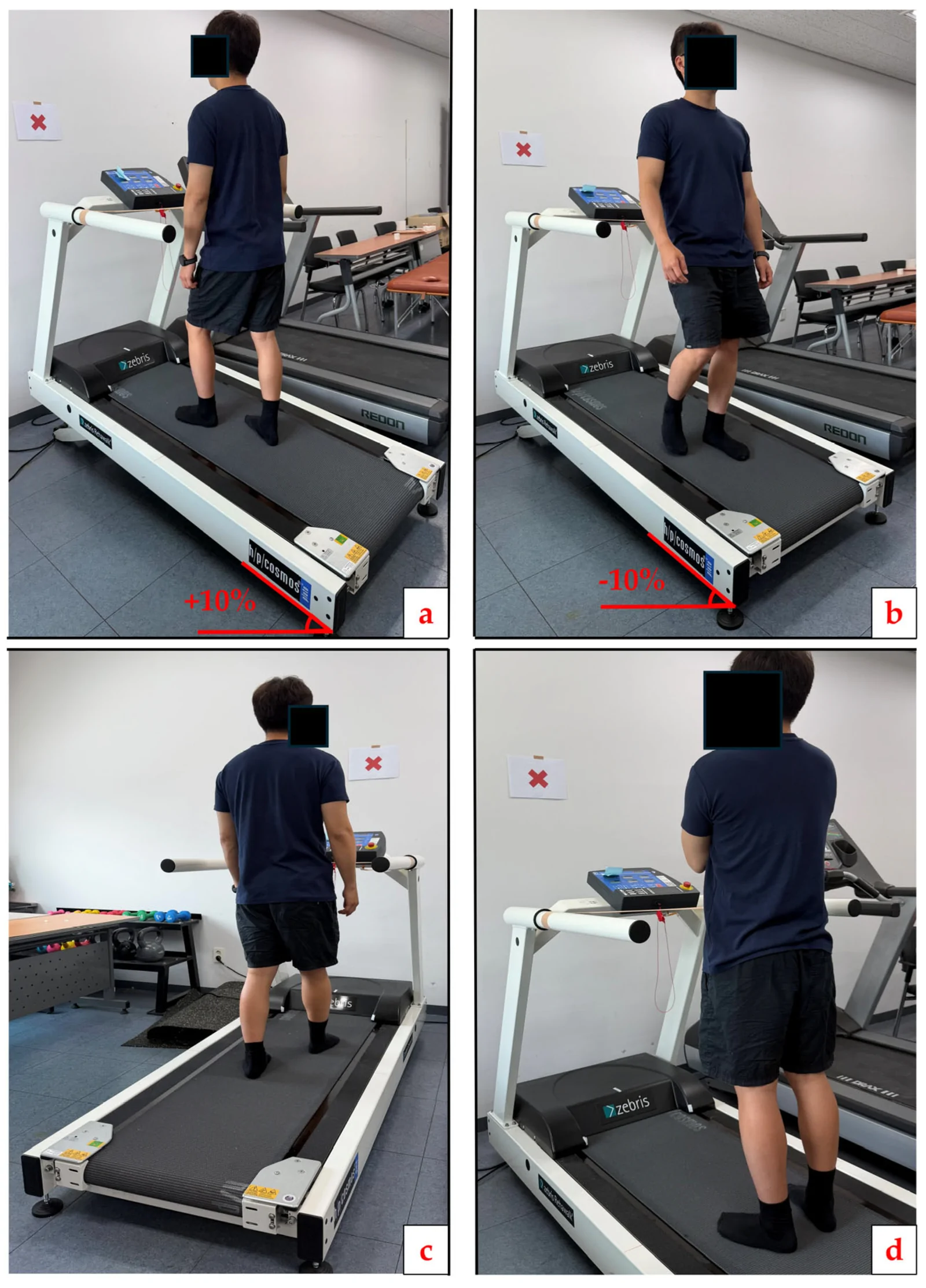
Experimental conditions on the instrumented treadmill. (**a**) Uphill walking at a +10% grade; (**b**) downhill walking at a −10% grade; (**c**) level walking at the preferred walking speed; (**d**) static balance assessment.

Participants then completed four locomotor conditions: (1) walking at their preferred walking speed; (2) running at 9 km/h; (3) uphill walking at a +10% grade; and (4) downhill walking at a −10% grade. The running speed was selected based on previous treadmill-running research in young adults [26]. Uphill and downhill walking were performed at +10% and −10% grades, respectively, consistent with previous treadmill-based gait research examining slope-dependent changes in healthy young adults [27]. The locomotor conditions are shown in Figure 1a–c.

Both graded walking conditions were performed at each participant’s preferred walking speed. Condition order was randomized with a predetermined permutation list to minimize potential order and fatigue effects. All 24 possible permutations of the four locomotor conditions were generated in advance, and one sequence was randomly assigned to each participant before testing. Each condition was performed for 2 min, with a 5-min rest interval between conditions. Participants were instructed not to hold the handrails during any condition, and an investigator remained beside the treadmill throughout to minimize the risk of falls. Static balance was assessed after completion of all locomotor trials, following a 5-min rest period.

### 2.4. Outcome Measures

The primary outcomes were the spatiotemporal gait parameters, and the secondary outcomes were the static balance measures.

#### 2.4.1. Spatiotemporal Gait Parameters

Spatiotemporal gait parameters were acquired using the pressure-sensor platform integrated into the instrumented treadmill (FDM-T; Zebris Medical GmbH, Isny, Germany) [21]. The pressure-sensing area measured 101.6 × 47.4 cm and contained 6720 capacitive pressure sensors at a density of 1.4 sensors/cm^2^. Data were sampled at 120 Hz and processed using Noraxon MR software (version 4.0.124; Noraxon, Scottsdale, AZ, USA). The temporal gait parameters included stance phase and single support phase. The spatial parameters included step length, stride length, and step width. The gait variables were operationally defined as follows. Stance phase was defined as the percentage of the gait cycle during which the foot was in contact with the ground. Single support phase was defined as the percentage of the gait cycle during which only the ipsilateral foot was in contact with the ground. Step length was defined as the distance between heel contact of one foot and heel contact of the contralateral foot, and step width as the distance between the right and left feet. Stride length was defined as the distance between two successive heel contacts of the same foot. Because participants in the FF group had flatfoot on only one side, gait variables were organized as involved and uninvolved sides rather than as anatomical left and right sides. In the FF group, the involved side was defined as the flatfoot side and the uninvolved side as the contralateral normal-foot side. Because all participants were right-leg dominant and the flatfoot was located on the left (non-dominant) side in 11 of the 14 participants in the FF group, the non-dominant and dominant sides of the control group were used as the corresponding involved and uninvolved sides, respectively. This approach was used to reflect the predominant combination of flatfoot side and limb dominance in the FF group. For the three participants with right-sided flatfoot, the right- and left-side gait data were recoded as involved and uninvolved, respectively, before statistical analysis.

Gait data were continuously recorded for 2 min under each locomotor condition. The first and final 30 s of each trial were excluded, and data from the middle 60 s were used for analysis. Gait events were automatically detected by the software based on the pressure-sensor data. Only complete steps within the selected 60-s analysis interval were included, and incomplete steps truncated at the beginning or end of the interval were excluded from the analysis. The same data-processing procedure was applied across all four locomotor conditions.

#### 2.4.2. Static Balance

Static balance was assessed using the pressure-sensor platform of the same instrumented treadmill with the treadmill belt stationary and the surface maintained at a 0% grade. Participants stood with their heels separated by 9 cm and their feet externally rotated by approximately 15° [28], with the standardized foot-position criteria applied to all participants. Foot positions were marked on the treadmill surface to ensure consistent placement across trials. Participants folded their arms across the chest and maintained their gaze on a stationary target positioned at eye level (Figure 1d). Center of pressure (COP) data were sampled at 120 Hz throughout each trial and processed in Noraxon MR software (version 4.0.124; Noraxon, Scottsdale, AZ, USA) using a low-pass filter with a cutoff frequency of 10 Hz [29].

Three 10-s trials of quiet standing were performed with the eyes open, with a 1-min rest interval between trials [30]. Before each trial, participants assumed the standardized standing position, and after postural stability was confirmed, a 5-s stabilization period was provided before data recording began. Trials were monitored by the examiner and were to be repeated in the event of loss of balance, foot movement, or other protocol deviation. No such events occurred during testing. The outcome measures included COP ellipse sway area, COP path length, and sway velocity. Sway area was calculated as the area of the 95% confidence ellipse enclosing the COP trajectory. COP path length was calculated as the total distance traveled by the COP during each trial, and sway velocity was calculated as COP path length divided by the trial duration. The mean of the three trials was used for analysis.

### 2.5. Statistical Analysis

Data were analyzed using SPSS Statistics version 25.0 (IBM Corp., Armonk, NY, USA). The distribution of continuous variables was evaluated with the Shapiro–Wilk test. Group comparisons for participant characteristics were performed using independent-samples *t*-tests when the data satisfied normality assumptions and Mann–Whitney U tests when they did not. Categorical variables were analyzed using the chi-square test.

Spatiotemporal gait outcomes were examined separately using 2 × 4 mixed-design repeated-measures analysis of variance (ANOVA), with group (flatfoot vs. control) entered as the between-subject factor and locomotor condition (walking, running, uphill walking, and downhill walking) as the within-subject factor. Box’s M and Levene’s tests were used to assess the equality of covariance matrices and error variances, respectively, and both assumptions were met for all outcome variables. Sphericity was evaluated with Mauchly’s test. When sphericity was violated, degrees of freedom were adjusted using the Huynh–Feldt correction for ε ≥ 0.75 and the Greenhouse–Geisser correction for ε < 0.75. Partial eta squared (η^2^_p_) was used to quantify effect size [31]. When the group × condition interaction was significant, simple-effects analyses were performed. The effect of locomotor condition was examined within each group, followed by Bonferroni-adjusted pairwise comparisons between conditions, whereas between-group differences were examined within each locomotor condition using Bonferroni-adjusted pairwise comparisons. Because the group factor comprised only two levels, these adjusted *p*-values were identical to the corresponding unadjusted values. The main effects of condition and group were also examined. Static balance variables were compared between groups using independent-samples *t*-tests for normally distributed outcomes and Mann–Whitney U tests for non-normally distributed outcomes. Statistical significance was defined as α = 0.05. Because no adjustment was made for multiple testing across outcome variables, the outcome analyses were considered exploratory.

## 3. Results

### 3.1. Participant Characteristics

**Figure 2:**
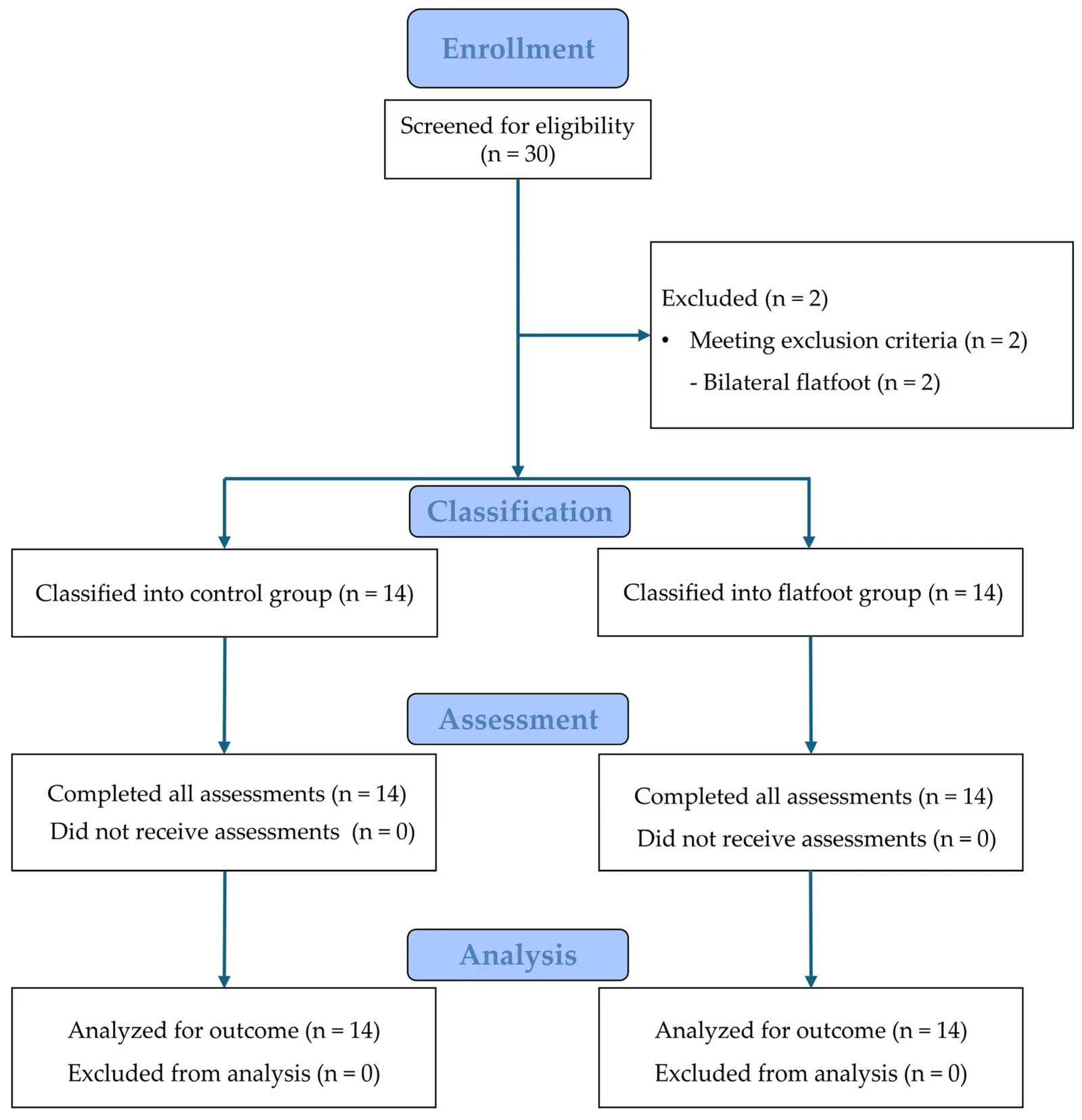
Flow diagram of the study.

A total of 30 individuals were screened for eligibility. Two individuals with bilateral flatfoot were excluded. The remaining 28 participants were classified into the flatfoot group (*n* = 14) or the control group (*n* = 14), and all completed the full protocol without dropout (Figure 2).

Participant characteristics are summarized in Table 1. The two groups did not differ significantly in age, sex, height, weight, body mass index, foot size, or preferred walking speed (*p* > 0.05). All participants reported right-leg dominance. The mean FPI-6 scores in the flatfoot group were 8.29 ± 1.98 on the involved side and 2.50 ± 1.74 on the uninvolved side. The observed FPI-6 scores ranged from 6 to 12 on the involved side and from 0 to 5 on the uninvolved side in the flatfoot group, and from −2 to 3 on the left foot and from −2 to 4 on the right foot in the control group. No participant in the control group had a highly supinated foot posture.

### 3.2. Temporal Gait Parameters

**Figure 3:**
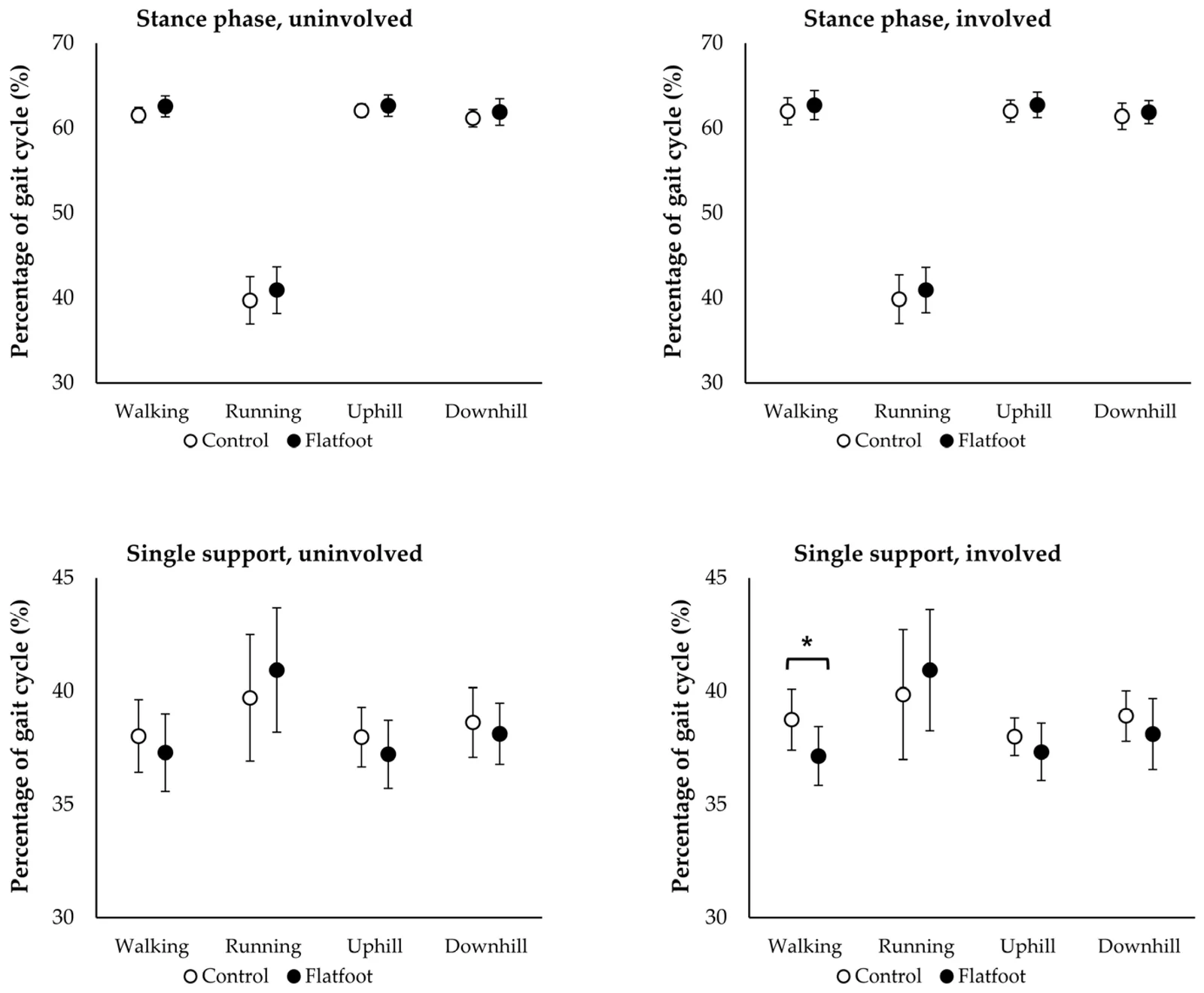
Temporal gait parameters across the four locomotor conditions in the control and flatfoot groups. Data are presented as mean ± standard deviation. * *p* < 0.05 for between-group comparisons within each condition, shown only for the variable with a significant group × condition interaction.

The temporal gait parameters are presented in Table 2 and Figure 3. The uninvolved stance phase showed no significant group × condition interaction (F(1.24, 32.19) = 0.211, *p* = 0.702, η^2^_p_ = 0.008). Significant main effects of group (F(1, 26) = 6.532, *p* = 0.017, η^2^_p_ = 0.201) and condition (F(1.24, 32.19) = 1198.203, *p* < 0.001, η^2^_p_ = 0.979) were observed, with a greater uninvolved stance phase in the flatfoot group when averaged across locomotor conditions. The involved stance phase also showed no significant group × condition interaction (F(1.24, 32.22) = 0.150, *p* = 0.755, η^2^_p_ = 0.006). A significant main effect of condition was observed (F(1.24, 32.22) = 1243.248, *p* < 0.001, η^2^_p_ = 0.980), whereas the main effect of group was not significant (F(1, 26) = 2.524, *p* = 0.124, η^2^_p_ = 0.088).

The uninvolved single support phase showed no significant group × condition interaction (F(1.25, 32.53) = 2.487, *p* = 0.118, η^2^_p_ = 0.087). A significant main effect of condition was observed (F(1.25, 32.53) = 17.876, *p* < 0.001, η^2^_p_ = 0.407), whereas the main effect of group was not significant (F(1, 26) = 0.141, *p* = 0.710, η^2^_p_ = 0.005). The involved single support phase showed a significant group × condition interaction (F(1.48, 38.47) = 3.827, *p* = 0.042, η^2^_p_ = 0.128). A significant main effect of condition was observed (F(1.48, 38.47) = 18.038, *p* < 0.001, η^2^_p_ = 0.410), whereas the main effect of group was not significant (F(1, 26) = 1.271, *p* = 0.270, η^2^_p_ = 0.047). The involved single support phase differed between groups only during preferred speed walking, with a greater percentage in the control group than in the flatfoot group (mean difference = 1.61 percentage points, 95% CI 0.58 to 2.63, *p* = 0.003), whereas no significant between-group differences were observed during running (mean difference = −1.08 percentage points, 95% CI −3.24 to 1.07, *p* = 0.312), uphill walking (mean difference = 0.67 percentage points, 95% CI −0.17 to 1.50, *p* = 0.112), or downhill walking (mean difference = 0.79 percentage points, 95% CI −0.26 to 1.85, *p* = 0.134). Within the control group, the involved single support phase was greater during downhill than uphill walking (*p* = 0.045), with no other significant pairwise differences (*p* > 0.05). Within the flatfoot group, the involved single support phase was greater during running than during preferred speed walking, uphill walking, and downhill walking (all *p* ≤ 0.004), and was greater during downhill than preferred speed walking (*p* = 0.039). No other pairwise differences were significant (*p* > 0.05).

### 3.3. Spatial Gait Parameters

**Figure 4:**
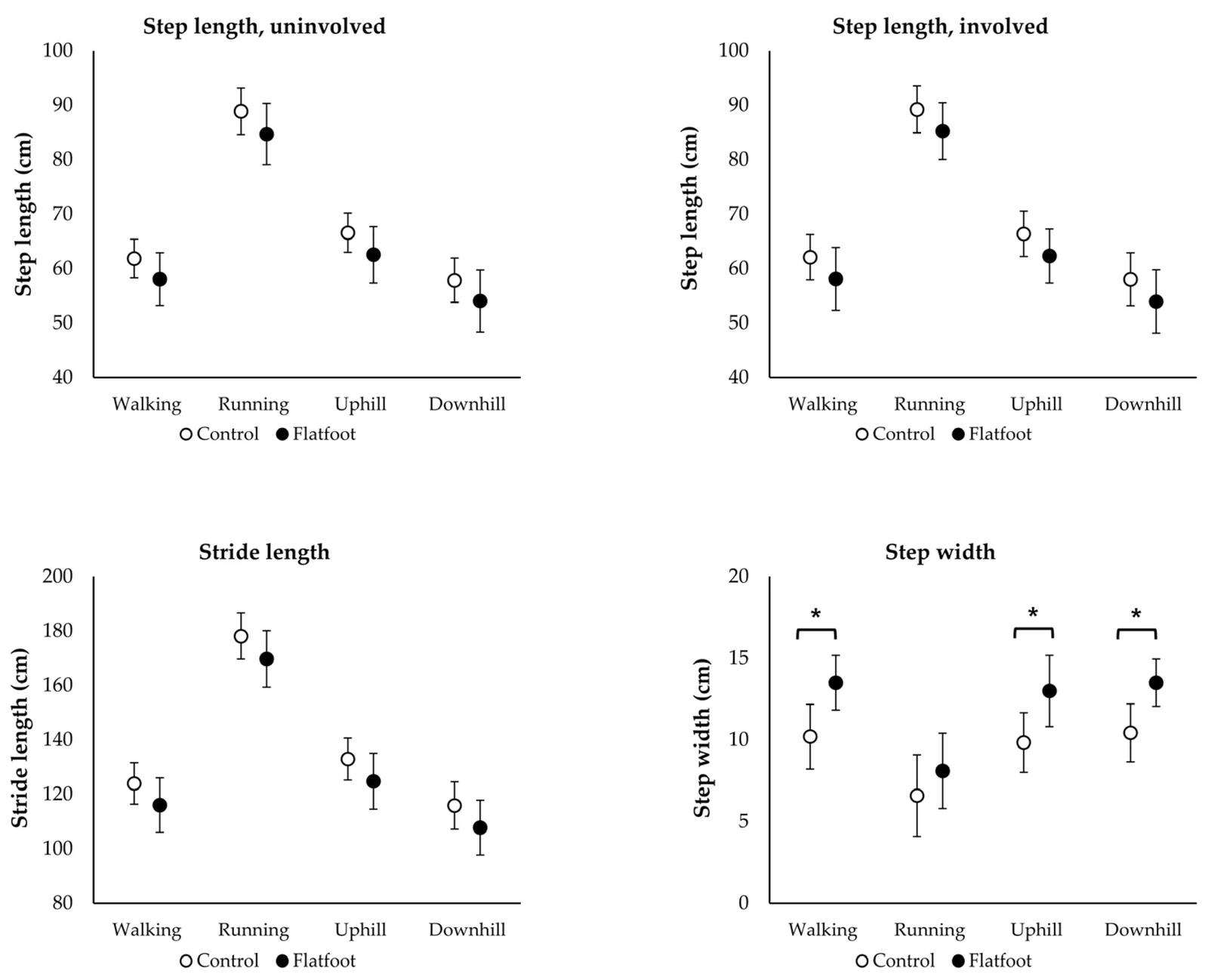
Spatial gait parameters across the four locomotor conditions in the control and flatfoot groups. Data are presented as mean ± standard deviation. * *p* < 0.05 for between-group comparisons within each condition, shown only for the variable with a significant group × condition interaction.

The spatial gait parameters are presented in Table 3 and Figure 4. The uninvolved step length showed no significant group × condition interaction (F(1.42, 36.80) = 0.027, *p* = 0.935, η^2^_p_ = 0.001). Significant main effects of group (F(1, 26) = 7.289, *p* = 0.012, η^2^_p_ = 0.219) and condition (F(1.42, 36.80) = 577.947, *p* < 0.001, η^2^_p_ = 0.957) were observed, with longer uninvolved step lengths in the control group when averaged across locomotor conditions. The involved step length showed no significant group × condition interaction (F(1.40, 36.40) = 0.002, *p* = 0.989, η^2^_p_ < 0.001). Significant main effects of group (F(1, 26) = 6.478, *p* = 0.017, η^2^_p_ = 0.199) and condition (F(1.40, 36.40) = 596.841, *p* < 0.001, η^2^_p_ = 0.958) were observed, with longer involved step lengths in the control group when averaged across locomotor conditions.

Stride length showed no significant group × condition interaction (F(1.62, 42.22) = 0.011, *p* = 0.978, η^2^_p_ < 0.001). Significant main effects of group (F(1, 26) = 7.171, *p* = 0.013, η^2^_p_ = 0.216) and condition (F(1.62, 42.22) = 798.932, *p* < 0.001, η^2^_p_ = 0.968) were observed, with longer stride lengths in the control group when averaged across locomotor conditions.

Step width showed a significant group × condition interaction (F(2.24, 58.24) = 4.738, *p* = 0.010, η^2^_p_ = 0.154). Significant main effects of group (F(1, 26) = 16.798, *p* < 0.001, η^2^_p_ = 0.392) and condition (F(2.24, 58.24) = 133.007, *p* < 0.001, η^2^_p_ = 0.836) were also observed. Step width was wider in the flatfoot group than in the control group during preferred speed walking (mean difference = 3.30 cm, 95% CI 1.88 to 4.72, *p* < 0.001), uphill walking (mean difference = 3.16 cm, 95% CI 1.60 to 4.72, *p* < 0.001), and downhill walking (mean difference = 3.07 cm, 95% CI 1.81 to 4.32, *p* < 0.001), whereas no significant between-group difference was observed during running (mean difference = 1.51 cm, 95% CI −0.36 to 3.39, *p* = 0.108). Within both groups, step width was narrower during running than during preferred speed walking, uphill walking, and downhill walking (all *p* < 0.001), whereas no significant differences were observed among the three walking conditions (all *p* > 0.05).

### 3.4. Static Balance

Static balance outcomes are presented in Table 4. Sway area did not differ significantly between the flatfoot and control groups (t = 0.518, *p* = 0.609). In contrast, COP path length was significantly greater in the flatfoot group than in the control group (z = −2.757, *p* = 0.005). Sway velocity was also significantly greater in the flatfoot group than in the control group (z = −2.757, *p* = 0.005). Because sway velocity was calculated as COP path length divided by the fixed 10-s trial duration, these two variables were mathematically dependent and yielded identical between-group statistical results.

## 4. Discussion

The FF group showed differences in gait characteristics compared with the control group, but these differences were confined to specific gait parameters and locomotor conditions. Inclined walking or running did not consistently magnify between-group differences. These condition-specific differences may partly reflect the different combinations of treadmill grade and locomotor speed across the experimental conditions.

### 4.1. Temporal Gait Parameters Across Locomotor Conditions

Temporal gait findings showed two patterns. The FF group demonstrated a greater uninvolved stance phase than the control group when averaged across locomotor conditions, whereas the involved single support phase was lower in the FF group only during preferred speed walking, indicating that less of the gait cycle was supported solely by the flatfoot limb under that condition. Because the single support phase of one limb corresponds closely to the swing phase of the contralateral limb, these two findings are biomechanically related and should not be interpreted as independent adaptations. However, the stance phase was numerically greater in the FF group on both sides, and differences between the two sides were not tested statistically. These findings therefore describe differences in support timing and do not demonstrate interlimb asymmetry or differences in limb loading. In addition, because the analytical sides were not completely matched with respect to limb dominance, the uninvolved-side finding should be interpreted cautiously. The medial longitudinal arch regulates foot compliance and energy storage across stance [32], and flat-arched feet exhibit altered multisegment foot motion in late stance [33]. Pan et al. reported interlimb differences in muscle-synergy organization during stance in individuals with unilateral flatfoot [16]. Whether these biomechanical or neuromuscular factors contributed to the lower involved single support phase during level walking cannot be determined from the present data.

Previous studies of flatfoot have reported temporal alterations that partly overlap with the present findings. Padrón et al. found greater total stance-phase contact time in adults with bilateral flatfoot [10]. The present findings are directionally consistent with this, as the FF group also showed a greater stance proportion on the uninvolved side, although the corresponding group difference was not statistically significant on the involved side. Although the specific support phase differed, Jiao et al. similarly reported a phase-specific temporal alteration, with a prolonged double-support phase despite no between-group differences in the overall proportions of stance or swing [34]. Together, these findings suggest that temporal differences associated with flatfoot may be evident in specific parameters or support phases rather than as a uniform pattern across all temporal measures. In contrast, Vijittrakarnrung et al. found no between-group differences in stance-phase proportion or other spatiotemporal parameters despite clear alterations in multisegment foot kinematics [9]. Their participants walked at a relatively fast controlled speed of 1.3–1.5 m/s, whereas the preferred walking speeds adopted in the present study were 1.18 and 1.12 m/s in the FF and control groups, respectively. Given that walking speed alters stance and double-support duration in healthy adults [35], this difference in gait protocol may partly explain the variability in temporal findings across studies.

The between-group difference in the involved single support phase was detected only during preferred speed walking. Because the conditions differed in grade, speed, and locomotor mode, this finding should be interpreted as condition-specific rather than as a response to increasing locomotor demand. Inclined walking requires adjustments in gait timing and lower-limb mechanics [13]. Running changes the temporal structure of gait by shortening the stance period and eliminating the double-support phase [36]. In addition, running was performed at a fixed speed of 9 km/h, whereas the walking conditions were performed at individually preferred speeds. The relative contribution of these task characteristics to the observed pattern cannot be determined from the present design.

### 4.2. Spatial Gait Parameters Across Locomotor Conditions

Spatial gait differences in FF were particularly evident in step width. Step width was wider in the FF group than in the control group during the three walking conditions, whereas the between-group difference was not statistically detected during running. Because the difference during running was in the same direction but the confidence interval included zero and was relatively wide, the non-significant result should be interpreted cautiously and may reflect limited precision associated with the small sample size. The mechanisms underlying the wider step width were not directly assessed in this study, and the interpretations below should be regarded as hypotheses. Mediolateral balance during walking requires active regulation through lateral foot placement [37,38], and step width is closely related to the control of mediolateral body motion [39]. The wider step width observed in the FF group may therefore reflect an adjustment in mediolateral foot placement. In pronated feet, imposed changes in step width have been shown to alter rearfoot eversion and medial longitudinal arch angle [40]. Shin et al. reported a narrower step width in individuals with flatfoot deformity [41], suggesting that differences in flatfoot severity and participant characteristics may partly explain the discrepancy. However, a structural contribution cannot be excluded, because flat-arched feet exhibit altered forefoot and rearfoot alignment during gait [33]. Whether the wider step width reflects an active foot-placement strategy or partly results from altered foot alignment would require concurrent kinematic assessment to determine.

Step length and stride length measures showed a different spatial pattern from step width. Both involved and uninvolved step lengths, as well as stride length, were shorter in the FF group when averaged across locomotor conditions. Because spatiotemporal parameters depend on locomotor speed, the possible influence of speed should be considered. Preferred walking speed did not differ significantly between the groups, although this does not establish equivalence in walking speed, and the group × condition interaction was not significant for these variables. The group means also showed the same direction of difference during running, in which all participants ran at the same fixed speed. These observations suggest that the shorter step and stride lengths may not be attributable solely to differences in walking speed, although an influence of walking speed cannot be completely excluded. During preferred speed walking, the stride lengths observed in the present study (1.16 m in the FF group and 1.24 m in the control group) were comparable to pooled estimates of 1.08 m and 1.22 m, respectively, reported in previous research [42]. Similar reductions in stride length have also been reported in the original studies contributing to this evidence, including cohorts with flatfoot and more advanced flatfoot deformity [41,43]. For step length, however, pooled evidence found no overall difference and substantial heterogeneity across studies [8]. The present finding of shorter step lengths on both the involved and uninvolved sides may therefore reflect differences in participant characteristics, foot-posture classification, or gait protocols across studies. Previous studies have also not consistently distinguished between unilateral and bilateral involvement, and flatfoot laterality may be a further source of variability in step-length findings. Because side-specific kinematics and kinetics were not measured, the mechanisms underlying the shorter step lengths observed on both sides cannot be determined from the present data.

### 4.3. Static Balance

The dissociation between area- and trajectory-based measures of postural sway is a notable aspect of the balance findings. In this study, the COP traveled a greater distance in the FF group, whereas a between-group difference in sway area was not statistically detected. Because sway velocity was derived from path length over the fixed trial duration, these two variables were mathematically dependent and should not be interpreted as independent findings. Descriptively, this pattern indicates greater COP movement without a detectable difference in sway area in the FF group. One possible explanation is that this reflects differences in postural adjustments during standing. However, COP trajectory measures do not directly quantify neuromuscular regulation, and this interpretation remains hypothetical. A structural contribution is plausible, as pronated foot structure may reduce the passive stability of the foot during weight bearing and has been associated with poorer standing postural control [44]. Impaired proprioceptive function may also contribute, as flatfoot has been associated with poorer ankle and knee joint position sense [20]. Faster postural sway during bipedal standing has likewise been reported in young adults with flatfoot [45]. The non-significant difference in sway area may reflect the relatively low postural demand of bipedal standing with the eyes open. Notably, postural-control differences associated with pronated foot structure have been demonstrated under the more demanding condition of single-limb stance with the eyes closed [44]. Whether a difference in sway area would be observed under more challenging postural conditions remains to be determined.

### 4.4. Limitations

Several limitations should be considered when interpreting these findings. First, the participants were young adults, the sample size was small, and the study was cross-sectional, limiting generalizability to other populations and precluding causal inference. In addition, multiple gait and balance outcomes were analyzed without multiplicity adjustment across outcome variables, and the findings should therefore be interpreted as exploratory. Second, FF was classified using the FPI-6, which characterizes static foot posture but does not assess arch flexibility or distinguish flexible from rigid flatfoot. Radiographic and detailed multisegment foot assessments were also not performed. Third, the absence of concurrent kinematic, kinetic, electromyographic, and plantar-pressure measures limits mechanistic interpretation of the observed gait and postural-control patterns. Fourth, gait was assessed on an instrumented treadmill, and the locomotor conditions differed in grade, speed, and locomotor mode, with each examined at only one level of task demand. Therefore, condition-dependent findings cannot be attributed to a single task characteristic. Fifth, static balance was assessed only during eyes-open bipedal standing. The absence of more challenging conditions, such as eyes-closed or single-limb standing, may have limited the ability to detect group differences in postural control. In addition, static balance was assessed after the locomotor trials, and although a 5-min rest period was provided, residual fatigue may have influenced the COP measurements because fatigue was not assessed. Finally, the involved limb was defined as the flatfoot side in the FF group and as the non-dominant side in the control group. Because three participants in the FF group had right-sided flatfoot and all participants were right-leg dominant, limb dominance and flatfoot involvement were not fully matched between groups, which may have influenced side-specific comparisons.

## 5. Conclusions

In young adults with unilateral FF, gait differences were observed across specific gait parameters and locomotor conditions rather than as a uniform pattern. When averaged across locomotor conditions, the FF group showed shorter step and stride lengths and a greater uninvolved stance phase. Step width was wider during the walking conditions, and COP path length was greater during static standing. Inclined walking and running did not consistently increase the between-group differences. These exploratory results suggest that considering multiple locomotor conditions and both limbs may provide additional descriptive information when characterizing gait in unilateral FF. However, the present study did not establish diagnostic or clinical benefit for any specific assessment strategy.

## Figures and Tables

**Table 1:** General characteristics of the participants.

Variable	Flatfoot (*n* = 14)	Control (*n* = 14)	*p*
Age (years)	21.71 ± 1.77	21.86 ± 1.41	0.815 ^b^
Sex (male/female)	8/6	7/7	1.000 ^a^
Height (cm)	170.71 ± 7.76	170.29 ± 7.66	0.886 ^b^
Weight (kg)	70.31 ± 13.81	67.89 ± 13.86	0.646 ^b^
Body mass index (kg/m^2^)	23.90 ± 2.89	23.23 ± 3.34	0.572 ^b^
Foot size (mm)	253.54 ± 15.00	245.36 ± 19.16	0.218 ^b^
Preferred walking speed (m/s)	1.18 ± 0.08	1.12 ± 0.18	0.260 ^b^
Flatfoot side (left/right)	11/3	—	—
Dominant leg (left/right)	0/14	0/14	—
Involved FPI-6 (score)	8.29 ± 1.98	—	—
Uninvolved FPI-6 (score)	2.50 ± 1.74	—	—
Dominant side FPI-6 (score)	—	1.50 ± 1.74	—
Non-dominant side FPI-6 (score)	—	1.21 ± 1.37	—

Data are presented as the mean ± standard deviation. FPI-6, Foot Posture Index-6. ^a^ Chi-square test; ^b^ independent-samples *t*-test.

**Table 2:** Comparison of temporal gait parameters across the four locomotor conditions.

Variable	Group	Walking (a)	Running (b)	Uphill (c)	Downhill (d)	Within- Group *p*	F	*p*	η^2^_p_
Stance phase, uninvolved (%)	Control	61.53 ± 0.88	39.71 ± 2.79	62.06 ± 0.79	61.16 ± 1.01	—	0.211	0.702	0.008
	Flatfoot	62.56 ± 1.22	40.93 ± 2.75	62.65 ± 1.25	61.89 ± 1.56	—			
Stance phase, involved (%)	Control	61.98 ± 1.58	39.85 ± 2.87	62.00 ± 1.29	61.38 ± 1.54	—	0.150	0.755	0.006
	Flatfoot	62.71 ± 1.71	40.93 ± 2.68	62.74 ± 1.49	61.88 ± 1.35	—			
Single support, uninvolved (%)	Control	38.02 ± 1.60	39.71 ± 2.79	37.97 ± 1.31	38.62 ± 1.54	—	2.487	0.118	0.087
	Flatfoot	37.29 ± 1.71	40.93 ± 2.75	37.22 ± 1.50	38.12 ± 1.35	—			
Single support, involved (%)	Control	38.75 ± 1.35	39.85 ± 2.87	37.99 ± 0.83 ^d^	38.91 ± 1.11 ^c^	0.012 *	3.827	0.042 *	0.128
	Flatfoot	37.14 ± 1.29 ^b,d^	40.93 ± 2.68 ^a,c,d^	37.32 ± 1.27 ^b^	38.11 ± 1.57 ^a,b^	<0.001 *			
Between-group *p*		0.003 *	0.312	0.112	0.134				

Data are presented as mean ± standard deviation. Temporal parameters are expressed as percentages of the gait cycle. Post hoc comparisons are shown only for the involved single support phase, for which the group × condition interaction was significant. η^2^_p_, partial eta squared. Involved, flatfoot side (flatfoot group) or non-dominant side (control group); uninvolved, contralateral side. ^a^, walking; ^b^, running; ^c^, uphill; ^d^, downhill conditions (*p* < 0.05). * *p* < 0.05.

**Table 3:** Comparison of spatial gait parameters across the four locomotor conditions.

Variable	Group	Walking	Running	Uphill	Downhill	Within- Group *p*	F	*p*	η^2^_p_
Step length, uninvolved (cm)	Control	61.85 ± 3.57	88.88 ± 4.29	66.58 ± 3.59	57.85 ± 4.07	—	0.027	0.935	0.001
	Flatfoot	58.06 ± 4.83	84.70 ± 5.61	62.56 ± 5.17	54.05 ± 5.69	—			
Step length, involved (cm)	Control	62.10 ± 4.17	89.24 ± 4.31	66.38 ± 4.16	58.05 ± 4.86	—	0.002	0.989	<0.001
	Flatfoot	58.10 ± 5.77	85.25 ± 5.19	62.33 ± 4.94	53.95 ± 5.81	—			
Stride length (cm)	Control	123.95 ± 7.61	178.12 ± 8.48	132.96 ± 7.67	115.90 ± 8.71	—	0.011	0.978	<0.001
	Flatfoot	116.06 ± 9.99	169.74 ± 10.37	124.79 ± 10.25	107.80 ± 10.06	—			
Step width (cm)	Control	10.20 ± 1.98 ^b^	6.59 ± 2.50 ^a,c,d^	9.84 ± 1.82 ^b^	10.43 ± 1.77 ^b^	<0.001 *	4.738	0.010 *	0.154
	Flatfoot	13.50 ± 1.67 ^b^	8.10 ± 2.31 ^a,c,d^	13.00 ± 2.18 ^b^	13.50 ± 1.44 ^b^	<0.001 *			
Between-group *p*		<0.001 *	0.108	<0.001 *	<0.001 *				

Data are presented as mean ± standard deviation. Post hoc comparisons are shown only for step width, for which the group × condition interaction was significant. η^2^_p_, partial eta squared. Involved, flatfoot side (flatfoot group) or non-dominant side (control group); uninvolved, contralateral side. ^a^, walking; ^b^, running; ^c^, uphill; ^d^, downhill conditions (*p* < 0.05). * *p* < 0.05.

**Table 4:** Comparison of static balance parameters.

Variable	Flatfoot (*n* = 14)	Control (*n* = 14)	t or z	*p*
Sway area (mm^2^) ^a^	214.65 ± 115.65	192.00 ± 115.95	t = 0.518	0.609
Path length (mm) ^b^	263.17 (229.97–310.20)	169.62 (101.07–219.13)	z = −2.757	0.005 *
Sway velocity (mm/s) ^b^	26.32 (23.03–31.02)	16.97 (10.10–21.90)	z = −2.757	0.005 *

Data are presented as mean ± standard deviation for normally distributed variables and median (interquartile range) for non-normally distributed variables. Each participant’s value represents the mean of three 10-s trials. ^a^, Independent *t*-test; ^b^, Mann–Whitney U test. * *p* < 0.05.

## Data Availability

The data presented in this study are available on request from the corresponding author due to ethical restrictions.

## Abbreviations

The following abbreviations are used in this manuscript:

ANOVA	Analysis of variance
BMI	Body mass index
COP	Center of pressure
FF	Flatfoot
FPI-6	Foot Posture Index-6
